# Combined RT-qPCR and pyrosequencing of a Spike glycoprotein polybasic cleavage motif can uncover pediatric SARS-CoV-2 infections associated with heterogeneous presentation

**DOI:** 10.1186/s40348-021-00115-x

**Published:** 2021-04-24

**Authors:** Patrick Philipp Weil, Jacqueline Hentschel, Frank Schult, Anton Pembaur, Beniam Ghebremedhin, Olivier Mboma, Andreas Heusch, Anna-Christin Reuter, Daniel Müller, Stefan Wirth, Malik Aydin, Andreas C. W. Jenke, Jan Postberg

**Affiliations:** 1 Clinical Molecular Genetics and Epigenetics, Faculty of Health, Centre for Biomedical Education & Research (ZBAF), Witten/Herdecke University, Alfred-Herrhausen-Str. 50, 58448 Witten, Germany; 2 HELIOS University Hospital Wuppertal, Children’s Hospital, Centre for Clinical & Translational Research (CCTR), Witten/Herdecke University, Heusnerstr. 40, 42283 Wuppertal, Germany; 3 HELIOS University Hospital Wuppertal, Institute of Medical Laboratory Diagnostics, Centre for Clinical & Translational Research (CCTR), Witten/Herdecke University, Heusnerstr. 40, 42283 Wuppertal, Germany; 4 HELIOS University Hospital Wuppertal, Experimental Pediatric Pneumology and Allergology, Children’s Hospital, Centre for Clinical & Translational Research (CCTR), Witten/Herdecke University, Heusnerstr. 40, 42283 Wuppertal, Germany; 5 Klinikum Kassel, Zentrum für Kinder- und Jugendmedizin, Neonatologie und allgemeine Pädiatrie, Mönchebergstr. 41-43, 34125 Kassel, Germany

**Keywords:** (+)RNA, COVID-19 surveillance, Pediatric SARS-CoV-2-associated diseases, Epidemiology

## Abstract

**Background:**

Reverse transcription of the severe acute respiratory syndrome coronavirus 2 (SARS-CoV-2) (+)RNA genome and subgenomic RNAs (sgRNAs) and subsequent quantitative polymerase chain reaction (RT-qPCR) is the reliable diagnostic gold standard for COVID-19 diagnosis and the identification of potential spreaders. Apart from clinical relevance and containment, for specific questions, it might be of interest to (re)investigate cases with low SARS-CoV-2 load, where RT-qPCR alone can deliver conflicting results, even though these cases might neither be clinically relevant nor significant for containment measures, because they might probably not be infectious. In order to expand the diagnostic bandwidth for non-routine questions, particularly for the reliable discrimination between negative and false-negative specimens associated with high C_T_ values, we combined the RT-qPCR workflow with subsequent pyrosequencing of a S-gene amplicon. This expansion can help to confirm SARS-CoV-2 infections without the demand of confirmative antibody testing, which requires to summon patients again for blood sampling few to several weeks after symptom onset.

**Results:**

We successfully established a combined RT-qPCR and S-gene pyrosequencing method which can be optionally exploited after routine diagnostics. This allows a reliable interpretation of RT-qPCR results in specimens with relatively low viral loads and close to the detection limits of qPCR. After laboratory implementation, we tested the combined method in a large pediatric cohort from two German medical centers (*n*=769). Pyrosequencing after RT-qPCR enabled us to uncover 5 previously unrecognized cases of pediatric SARS-CoV-2-associated diseases, mainly exhibiting mild and heterogeneous presentation—apart from a single case of multisystem inflammatory syndrome in children (MIS-C) associated with SARS-CoV-2, who was hospitalized in the course of the study.

**Conclusions:**

The proposed protocol allows a specific and sensitive confirmation of SARS-CoV-2 infections close to the detection limits of RT-qPCR. The tested biotinylated primers do not negatively affect the RT-qPCR pipeline and thus can be optionally applied to enable deeper inspection of RT-qPCR results by subsequent pyrosequencing. Moreover, due to the incremental transmission of SARS-CoV-2 variants of concern, we note that the used strategy can uncover (Spike) P681H allowing the pre-selection of SARS-CoV-2 B.1.1.7 candidate specimens for deep sequencing.

**Supplementary Information:**

The online version contains supplementary material available at 10.1186/s40348-021-00115-x.

## Background

**Fig. 1:**
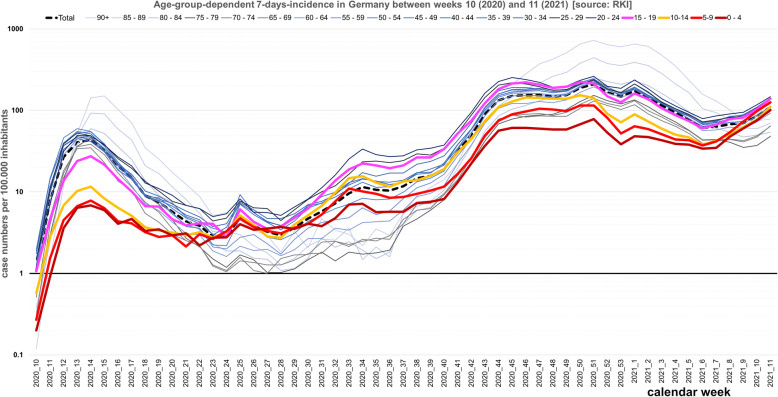
Seven-day incidence development in Germany between calendar weeks 2020_10 and 2021_11. According to the laboratory-confirmed SARS-CoV-2 case numbers continuously reported by the Robert Koch Institute (www.RKI.de), the 7-day incidence for pediatric infections increased very reminiscent of most other age groups or even faster approx. since calendar weeks 2021_6/7. At least for the age groups between 0 and 14 years, this development contrasts the spring situation, when these children were less affected than other age groups

As of April 26, 2020, at Helios University Hospital Wuppertal during a period of attenuation which followed the first peak phase of SARS-CoV-2 transmission in Germany, only 2.3% of all laboratory-confirmed SARS-CoV-2-positive cases were children or teenagers (age group 0–19 years). Nationwide, as of September 8, 2020, the contribution of this age group to all SARS-CoV-2-positive cases had increased to 10.5% [1]. This development was in agreement with observations made globally, in particular, in the USA, whose population remains among the most affected by the SARS-CoV-2 pandemic. Since then, a trend of weekly median age decline for persons with COVID-19-like illness was reported for an observation period between May 3 and August 29, 2020. In particular, a steady increase of the percentage fraction of all confirmed SARS-CoV-2-positive cases for the 0–19-year-olds was observed in the USA starting from 7.4% in May, 10.8% in June, 14.0% in July, and preliminary being elevated up to 15.5% in August 2020 [2]. Reminiscent of this, at least since early autumn 2020, the 7-day incidence for all pediatric age groups increased in similar ways as observed for most other age groups in Germany or apparently even faster since 2021 calendar weeks 6–7 (Fig. 1). As evidence is accumulating that pediatric presentation of SARS-CoV-2-associated diseases can possibly be more heterogeneous than adult COVID-19 [3–5], there is an urgent need to recognize the full spectrum of unusual pediatric SARS-CoV-2-borne diseases. Here, we report a field test on a pediatric cohort as well as a detailed protocol for an expanded SARS-CoV-2 (+)RNA detection method, which relies on the complementary exploitation of RT-qPCR and pyrosequencing of a genome fragment encoding the SARS-CoV-2 Spike glycoprotein polybasic cleavage motif. Following rigorous implementation, we applied the test on two pediatric cohorts from two German medical centers (Helios University Hospital Wuppertal, North Rhine-Westphalia, West Germany, and Klinikum Kassel, Hessen, Central Germany) comprising 769 children in total (*n*=599 [Wuppertal]; *n*=170 [Kassel]; 44% female/56% male; 53.6% 0–5 years, 28.7% 6–12 years, 17.7% 13–17 years). Specifically, all pediatric outpatients as well as hospitalized pediatric patients with fever or respiratory symptoms (age between day 1 after birth and >18 years) were included, from whose legal guardians we obtained written informed consent. Retrospectively, apart from a single hospitalized case who developed multisystem inflammatory syndrome in children (MIS-C) associated with SARS-CoV-2 (briefly described below) in the course of the study period, none of the patients fulfilled indications for SARS-CoV-2 testing, which would have been arisen if there was a sufficient clinical picture and/or an epidemiological connection to an infectious event or a risk group. In order to conduct as many and as targeted tests as possible, these indications are continuously adapted to the course of the pandemic and published by the German Federal Ministry of Health (“National Teststrategie”; www.bundesgesundheitsministerium.de) and the Robert-Koch-Institute (www.RKI.de).

The rationality of a combined RT-qPCR/pyrosequencing approach is based on the possibility to confirm SARS-CoV-2 infections associated with low viral load without the demand of confirmatory antibody testing, which requires to summon patients again for blood sampling, since the antibody response emerges typically few to several weeks after symptom onset [6].

This optional expansion can thus contribute to the rate reduction of uncertain qPCR results in specific cases, which can be caused by low viral loads and also might be influenced by several confounding factors [7], thus occasionally leading to false-negative qPCR results, particularly when PCR is close to its detection limits. That this matters, is substantiated by studies of Arnaout and co-workers, who reported very recently that the limit of detection between currently approved assays can vary over 10.000-fold [8], potentially causing substantial assay-dependent false-negative rates. Theoretically, such false-negative results associated with very low viral loads could be tolerable with respect to containment measures for individuals who do not transmit the virus. But a study on a Wanzhou District cohort suggests that SARS-CoV-2-infected individuals with subclinical courses or even without any symptoms can nevertheless shed the virus, and their viral loads as estimated by C_T_ values (IQR_Orf1b[asymptomatic]_ 30.9–35.8|IQR_Orf1b[symptomatic]_ 30.3–35.1, *p*=0.336; IQR_N[asymptomatic]_ 29.5–34.6|IQR_N[symptomatic]_ 31.3–37.2, *p*=0.126) seemed to be similar to symptomatic patients [9]. Whereas measurable virus shedding is not equivalent with viral infectivity, there is a good correlation between RT-qPCR C_T_ value and SARS-CoV-2 culture positivity with higher C_T_ values correlating with declining culturability and presumably infectiousity [10]. Even though in that study, virus culturing from approx. 8% of specimens with C_T_>35 was successful, demonstrating that it can be a balancing act to categorize negative and false-negative in case of high C_T_ values. Here, we demonstrate that RT-qPCR primer biotinylation enables the option of reinvestigation of selected RT-qPCR results by pyrosequencing without disturbing the routine RT-qPCR workflow.

## Results

### Design and implementation of a combined SARS-CoV-2 RT-qPCR/pyrosequencing test to complement established diagnostic workflows

**Fig. 2:**
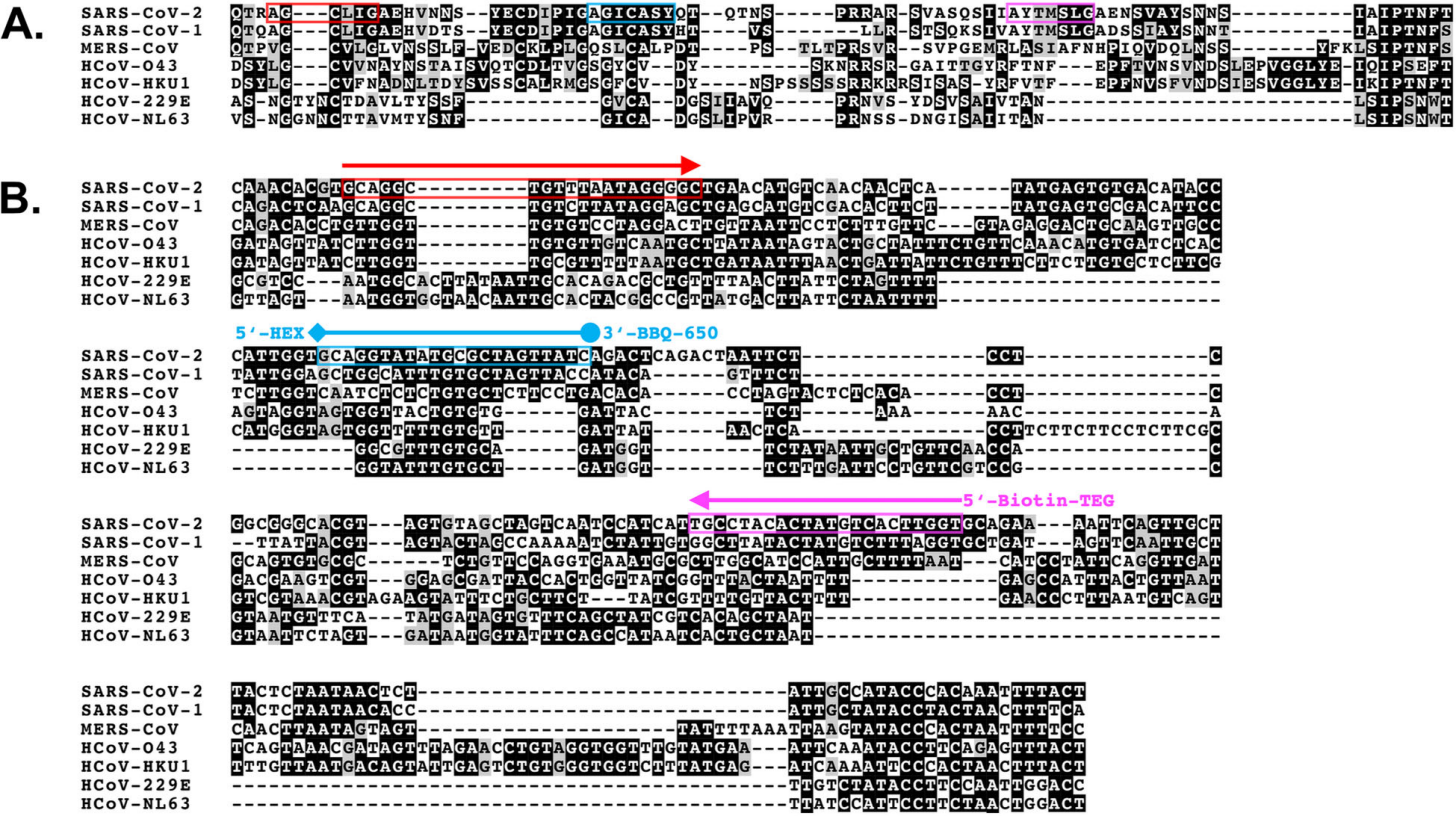
Comparison of homologous protein sequence segments (**a**) and corresponding cDNA segments (**b**) between human coronaviruses. **a** We applied the Clustal *W* algorithm of MEGA [13] to conduct multiple-sequence alignments using the translated protein sequences of the SARS-CoV-2 Spike (S) glycoprotein and homologous S protein sequences from other human coronaviruses. Here, a segment harboring polybasic cleavage motif (Q644 to T720 with respect to SARS-CoV-2 S protein). The highlighted residues are conserved in most human coronavirus (black shaded) or are similar between some human coronaviruses (gray shaded). The colored boxes are framing residues, which correspond to the target position of tested oligonucleotides: forward primer (red), probe/sequencing primer (blue), reverse primer (magenta). **b** The aligned protein sequences were backtranslated into the encoding cDNA sequences. Similarly, as described above for protein sequences, black or gray shading was used to illustrate identical or similar nucleotide positions. For combined RT-qPCR and pyrosequencing, we selected the following marked sequences for oligonucleotide design: 1. forward primer (red box/arrow); 2. TaqMan probe with 5′-HEX and 3′-BBQ-650 modifications (blue box/line); 3. sequencing primer without end-modification (blue box—the same sequence as TaqMan probe); 4. reverse primer with 5′-Biotin-TEG (magenta box/arrow)

We targeted a region encoding a polybasic cleavage motif within the SARS-CoV-2 Spike glycoprotein (S). Multiple sequence alignment analyses demonstrate that this region could be a suitable differentiator between SARS-CoV-2, related coronaviruses from several animal hosts [11], and SARS-CoV-1, MERS-CoV, and other HCoVs (Fig. 2a). For RT-qPCR, we selected a set of forward primer (S_pbc_-CoV-2-F: 5′-GCAGGCTGTTTAATAGGGGC-3′) and 5′-biotinylated reverse primer (S_pbc_-CoV-2-R_BIO_: 5′-biotin-TEG-ACCAAGTGACATAGTGTAGGCA-3′), since Primer-BLAST (https://www.ncbi.nlm.nih.gov/tools/primer-blast/index.cgi) query confirmed the probable suitability of the selected primer set predicting a 162-bp SARS-CoV-2-specific amplicon matching perfectly to the complete reported list of SARS-CoV-2 isolates. As a probe for TaqMan qPCR, we used 5′-HEX-ATTGGTGCAGGTATATGCGCTAGTTATC-BBQ-650-3′ (S_pbc_-CoV-2-P). Without carrying the 5′-/3′-modifications, we selected the same oligonucleotide sequence for the pyrosequencing primer (S_pbc_-CoV-2-S) (Fig. 2b; Table S1).

During method establishment, we made use of mostly adult specimens, which underwent routine COVID-19 diagnostic RT-qPCR testing and were pre-categorized “(RT-qPCR)-confirmed SARS-CoV-2-positive” or “negative”. For comparison, we used the full set of “Charité protocol” amplicon targets (RdRP, E, N) [12] (Table S1). Before RT-qPCR, the total RNA was purified from 250 μL of nasopharyngeal swabs or bronchoalveolar lavage specimens using the guanidinium isothiocyanate (GITC) extraction method. Besides, due to the RT-qPCR reagent manufacturer’s recommendation, we tested whether the RNA purification step could be dispensable. Briefly, using stabilized raw specimens from SARS-CoV-2 as RT-qPCR templates, results from the same specimens using purified RNA could only be matched in some cases (Figure S1). Therefore, we neglected this approach during our successive experiments.

**Fig. 3:**
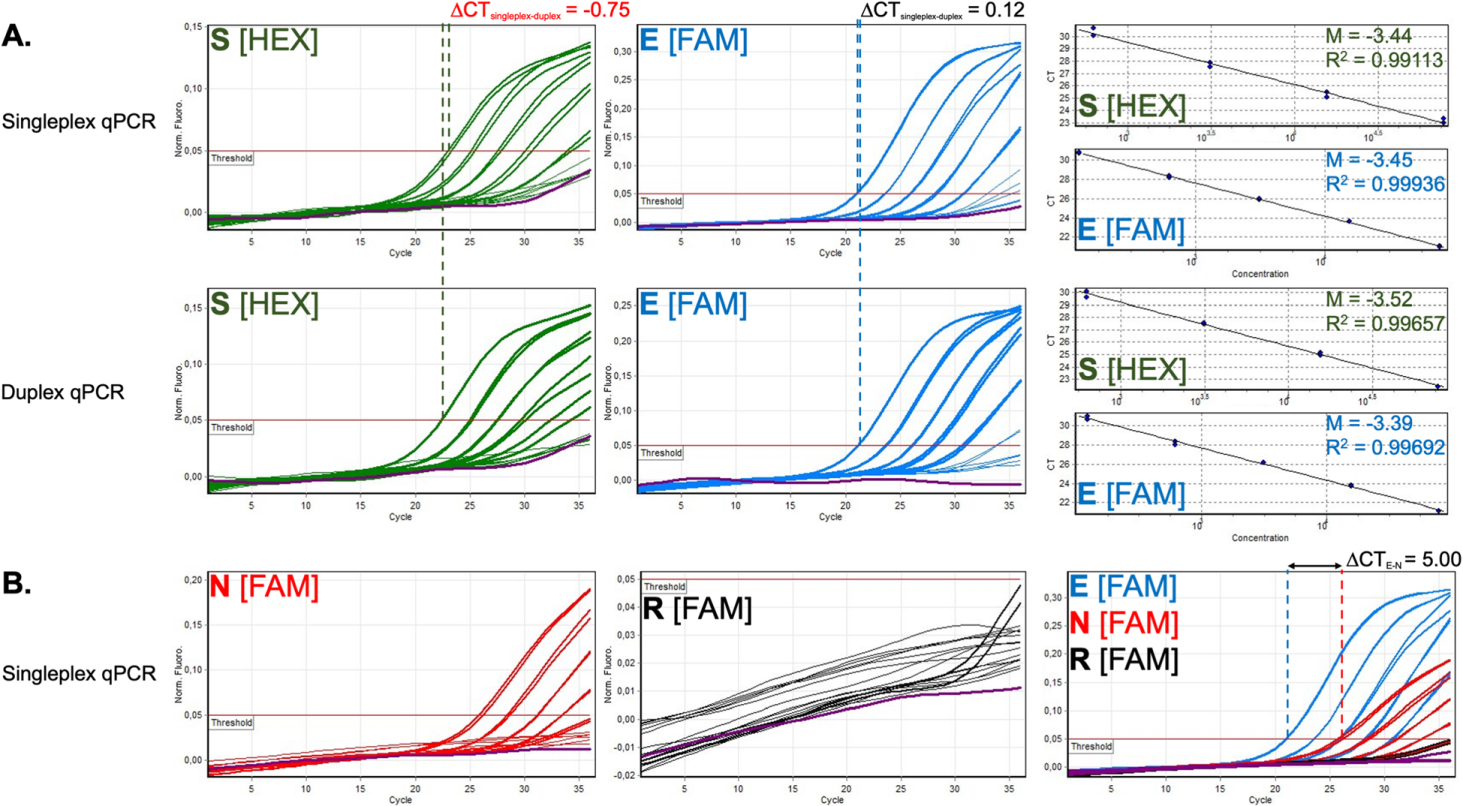
Comparative characterization of RT-qPCR efficiency and sensitivity. The same clinical specimen was used for all tests. **a** The S-gene amplicon (S) was compared with ORF E (E) in singleplex and duoplex reactions. **b** The direct performance comparison of ORF E (E) amplicons with ORF N (N) and RdRP (R) demonstrated the differences in sensitivity, whereby E>N>R

Reverse transcription and subsequent amplification of cDNA were carried out using universal one-step RT-qPCR reaction mastermixes. A detailed protocol including recommended reagents is provided in the “Methods” section. Quantitative PCR programs often use an excess of cycling steps, frequently leading to the incremental enrichment of unspecific byproducts during later cycles. With respect to the quality of subsequent pyrosequencing, we aimed at the optimization of PCR cycle numbers for singleplex, duoplex, and triplex PCR approaches. We determined that 36 sequential qPCR cycles of denaturation, annealing, and elongation were a good compromise with respect to multiple prudential reasons: (1) Typically, for different amplicons, we observed that the cycle threshold (C_T_) limit for the faithful discrimination between positive and negative samples was below a C_T_ of 35 (Fig. 3). Consistently, semi-quantitative PCR and subsequent electrophoresis resulted in a specific 162-bp band and no or occasionally few weak byproducts (Figure S2A). (2) For approx. 35 or more cycles, we observed accelerated curve increments and thresholds crossing also for negative samples and no-template controls indicating ongoing amplification of PCR byproducts. Using an excess of PCR cycles, multiple unspecific byproducts and DNA smear of higher molecular weight are observable in confirmed positive and negative specimens (Figure S2B). Besides an excess of PCR cycles, we identified multiplex PCR approaches where more than one amplicon is targeted by multiple primer sets in one PCR reaction as another possible confounding factor influencing PCR quality and successive pyrosequencing. To test this hypothesis, we performed triplex RT-qPCR reactions by simultaneously using RdRP, Orf E, and Orf N amplicon targets. Semi-quantitative analyses by agarose gel electrophoresis demonstrate that numerous low- and high-molecular-weight byproducts were amplified in both cases when RNAs from confirmed SARS-CoV-2-positive specimens or negative specimens were used (Figure S3). Since we assumed that biotinylated RT-qPCR byproducts could massively impair successive pyrosequencing, we decided to neglect triplex approaches for the intended combinatorial use. With an emphasis on the SARS-CoV-2 S-gene amplicon, we instead aimed to compare the specificities, efficiencies, and sensitivities of singleplex RT-qPCR vs. duoplex RT-qPCR. In order to further characterize the performance of the SARS-CoV-2 S-gene amplicon target and to select the best additional amplicon for a duoplex approach, we performed comprehensive RT-qPCR tests using the S-gene, Orf E, Orf N, and RdRP as amplicon targets and using serially diluted specimens from the same RT-qPCR-confirmed SARS-CoV-2-positive specimens for all RT-qPCR reactions. Figure 3 illustrates the performance of different qPCR assays for one representative SARS-CoV-2-positive nasopharyngeal specimen. Taken together, TaqMan RT-qPCR for both the S-gene amplicon and the Orf E amplicon with similar sensitivity was indicated by low ΔC_T_ between S-gene and Orf E in singleplex reactions. Further, we deduced from a series of serial template dilutions that amplification efficiency for both the S-gene target and the Orf E target is high but weakens between C_T_=30 and C_T_=35. In duoplex RT-qPCR assays targeting simultaneously the S-gene target and Orf E in one reaction, no notable changes in sensitivity or qPCR efficiency were seen (Fig. 3a). With respect to the efficiency, also, the Orf N amplicon target performed well, but its sensitivity lagged behind as indicated by a large ΔC_T_ when compared to Orf E (Fig. 3b). In stark contrast, the RdRP amplicon frequently performed volatile and appeared to lag behind the sensitivities of all other amplicons in the majority of specimens examined—as in the illustrated case, where even the curve for the undiluted sample did not cross the predefined threshold (Fig. 3b). Therefore, apart from a singleplex S-gene RT-qPCR/pyrosequencing approach, we considered a duoplex (S-gene and Orf E) RT-qPCR/S-gene pyrosequencing approach as a promising combination for the faithful detection of active SARS-CoV-2 infections.

**Fig. 4:**
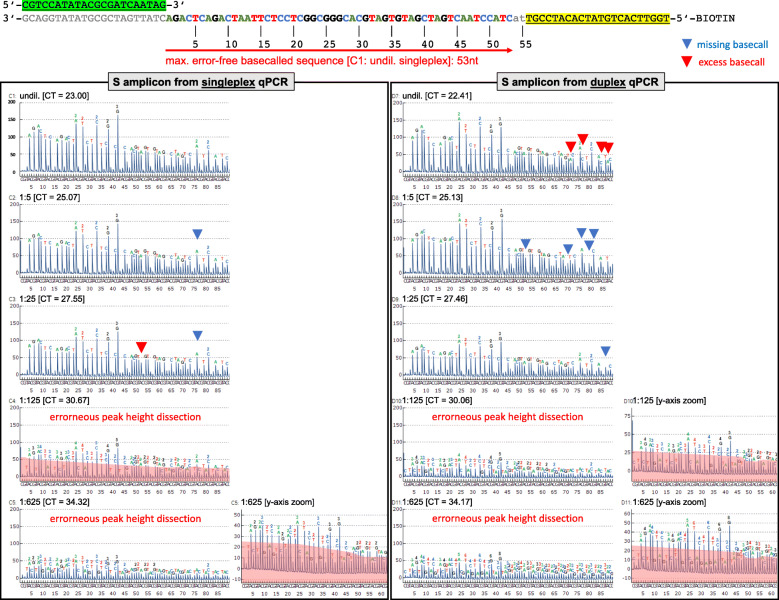
Comparative characterization of pyrosequencing sensitivity and basecalling quality. Serial dilutions of the same clinical specimen were used for all tests. Top: targeted region and principle of S-gene fragment pyrosequencing. Limited by the used PCR primers, the maximum theoretical sequence length is 55 nt. Bottom left: resulting pyrograms from serial template dilutions used for RT-qPCR in singleplex reactions and subsequent pyrosequencing of the S-gene amplicons using the antisense single-strand (as defined by the biotinylated reverse primer [S_pbc_-CoV-2-R_BIO_]) are shown. From the undiluted singleplex reaction, we obtained the longest unbiased SARS-CoV-2-specific sequence fragment, which had an error-free length of 53 nt. Bottom right: resulting pyrograms from serial template dilutions used for RT-qPCR in duoplex reactions and subsequent pyrosequencing of the S-gene amplicons using the antisense single-strand (as defined by the biotinylated reverse primer [S_pbc_-CoV-2-R_BIO_]) are shown. From the undiluted duoplex reaction, we obtained a maximum unbiased SARS-CoV-2-specific sequence fragment of 44 nt in length. Bottom left/right: the associated C_T_ values are shown beside the degree of dilution. Lower template concentrations led to the occasional occurrence of miscalled bases (missing bases: red triangle; excess bases: blue triangle) and gradual convergence of signal and noise peaks. Whereas concomitantly, automated basecalling gradually failed to separate signal from noise, the SARS-CoV-2 specific sequence could be identified by manual inspection much longer

To test this hypothesis, we performed subsequent pyrosequencing of the biotinylated single-stranded S-gene amplicon using the same serial dilution samples described above (Fig. 4). The proposed pyrosequencing approach could theoretically allow to read a SARS-CoV-2 (+)RNA-specific sequence fragment of 55 nt. Using the undiluted sample from the singleplex S-gene amplicon RT-qPCR reaction, we achieved an unbiased 53-nt sequence fragment, which could unambiguously be assigned to the SARS-CoV-2 reference genome. Successive serial dilutions led to modest enrichment of miscalled bases in the resulting pyrogram but still allowed the faithful assignment of the called sequence to SARS-CoV-2 up to a corresponding approx. C_T_≈30. Serial dilutions were gradually accompanied by a weakening of pyrogram-signal strength and a decreased signal-to-noise ratio. Automated basecalling of the SARS-CoV-2 sequence failed frequently for samples with C_T_≫30. However, for a range between C_T_≈30 and C_T_≈35, the most adverse reason counteracting SARS-CoV-2 sequence recognition appeared to be the decremental signal-to-noise ratio, which led to incremental false-positive calling of repetitive bases. The illustrated examples suggest the presence of signals encoding the targeted SARS-CoV-2, which could be separable from the threshold noise signals. S-gene pyrosequencing performed equally well, when a duoplex (S-gene and Orf E) RT-qPCR was conducted priorly (Fig. 4). We noted a tendency of slightly increasing numbers of missing or excess basecalls towards the 3′-end of the pyrosequenced SARS-CoV-2 fragment. As a preliminary conclusion, we highlight that the proposed pyrosequencing approach does not negatively affect the preceding RT-qPCR pipeline. Second, for selected cases, it adds important value to RT-qPCR, where this method alone delivers conflicting results. This can happen particularly close to the detection limits qPCR (C_T_ values ≫30). Figure S4 shows an example where RT-qPCR occasionally resulted in an overlap of curves from SARS-CoV-2-positives and negatives. Some curves from negative samples crossed the threshold within a range between C_T_≈30 and C_T_≈35, which complicated the reliable discrimination between PCR-positives, PCR-negatives, and PCR false-negatives. In such cases, pyrosequencing can help to confirm the RT-qPCR results. In the next step, after protocol implementation, we checked the sustainability of the combined RT-qPCR/pyrosequencing method in a field test.

### Performance of the combined methods of RT-qPCR and pyrosequencing in a field study on pediatric patients from two German medical centers

We tested our combined RT-qPCR/pyrosequencing method on two pediatric cohorts from two German medical centers comprising 769 undiagnosed children in total (*n*=599 [Wuppertal]; *n*=170 [Kassel], who did not fulfill the criteria for routine SARS-CoV-2 testing at day of presentation. Initial screening of all specimens was done by RT-qPCR. Only, RT-qPCR-positive samples or samples with a suspicious curve shape were reanalyzed by pyrosequencing. Besides the S-gene amplicon, we used at least ORF E as backup amplicon as well as ORF N and RdRP in some cases. We observed that even for RT-qPCR results with relatively high C_T_ values, successive pyrosequencing could unambiguously confirm SARS-CoV-2 infections. In some cases, we obtained patients’ agreement to investigate the SARS-CoV-2-specific antibody response. In sum, using retrospectively the combined RT-qPCR/pyrosequencing method, we confirmed 5 pediatric cases of SARS-CoV-2-associated diseases among the entire cohort exhibiting heterogenous presentation (Table S2). It is noteworthy that in contrast to these uncovered cases, only a single case of multisystem inflammatory syndrome in children (MIS-C) associated with SARS-CoV-2 was hospitalized in the course of the study. This patient, who underwent routine laboratory testing, is included in the overview for comparison (brief case report 6). We note that the patients presented here can serve as examples of the application of the diagnostic procedure of combined RT-qPCR/pyrosequencing, but due to the limited cohort size, our results might not necessarily indicate evidence-based general usefulness of this approach.

#### Brief case report 1: Adolescent male with sore throat (age group 13–17 years)

During the 2020 spring peak of incremental SARS-CoV-2 transmission at the end of April, an adolescent male patient presented at the emergency department in slightly reduced general condition with 37.5°C body temperature and reddened throat. The clinical examination was otherwise completely normal. At time of presentation, the patient did not fulfill the criteria for routine SARS-CoV-2 testing. The specimen was incidentally tested and classified potentially being SARS-CoV-2-positive in the course of our serial examination of the Wuppertal pediatric cohort using the newly developed S-gene amplicon target for singleplex RT-qPCR (repetitive C_T_ values 25.47 and 25.33). The result was unambiguously confirmed by pyrosequencing (Figure S5) and backed-up by positive RT-qPCR results using the ORF E (C_T_ value 23.78), ORF N (C_T_ value 27.47), and RdRP (C_T_ value 31.23) amplicon targets. Three months after the first presentation, the patient was positive for SARS-CoV-2-specific antibodies (34.20+; Roche), strongly suggesting that the boy underwent an inconspicuous course of SARS-CoV-2 infection.

#### Brief case report 2: Pre-school boy with high-grade fever (age group 4–6 years)

A pre-school boy with short bowel syndrome presented at the pediatric emergency department with a history of 1-day high-grade fever. Clinical examination did not reveal a specific focus. Since he had a central venous catheter (CVC) via the right internal jugular vein for partial parenteral nutrition, he was admitted for suspected central line sepsis and started on intravenous antibiotics. After 1 day, the condition of the patient and the inflammatory markers improved. The blood culture was positive for *Klebsiella pneumoniae*. On day 2 of inpatient care, the patient developed facial, neck and, upper limb edema, vein distention in the upper chest, and shortness of breath suggestive of superior vena cava (SVC) syndrome. CT angiography of the chest confirmed this diagnosis demonstrating a stenosis of the SVC (lumen diameter 1.4 mm), adjacent thrombosis, and multiple collateral veins draining towards an enlarged vena azygos. The mean C_T_ value of repetitive RT-qPCR was relatively high (C_T_ 34.06) with a conspicuous curve shape. SARS-CoV-2 was unambiguously confirmed by pyrosequencing (Figure S5).

#### Brief case report 3: Male secondary school child with isolated swollen neck lymph node (age group 10–12 years)

A male prepubescent secondary school child presented to the emergency department in late March 2020 complaining about a “bump” behind his left ear. Otherwise, he was completely asymptomatic. Clinical examination revealed a non-tender swelling behind the left ear of about 7–8 mm in diameter which was interpreted as an enlarged lymph node as well as slightly enlarged cervical lymph nodes on the same site. No cardiac, neurological, pulmonary, or gastrointestinal abnormalities were noted, and the patient was sent home. At the time of presentation, the patient did not fulfill the criteria for routine SARS-CoV-2 testing. The mean C_T_ value of repetitive RT-qPCR was relatively high (C_T_ 35.56) with a conspicuous curve shape. SARS-CoV-2 was unambiguously confirmed by pyrosequencing (Figure S5).

#### Brief case report 4: Female adolescent with EBV-like disease (age group 14–17 years)

A female adolescent presented late in March 2020 with a high fever up to 40.2°C, hepatomegaly, and tonsillitis. She was found to have acute Epstein-Barr virus infection and was discharged home 3 days later without fever in good general condition. Nine days later, she presented again with low temperature up 38.4°C, general malaise, and slightly increased liver enzymes. All other diagnostic tests at that time—including abdominal ultrasound, laboratory studies, and urine analysis—were normal. During the following 2 days, she improved spontaneously and was discharged home. At that time, symptoms were believed to be associated with the recent EBV infection. No SARS-CoV-2 testing was performed since the patient did not fulfill the clinical criteria for suspected COVID-19 at that time. The mean C_T_ value of repetitive RT-qPCR was relatively high (C_T_ 32.80) with a conspicuous curve shape. SARS-CoV-2 was unambiguously confirmed by pyrosequencing (Figure S5). In October 2020, the patient was found to have IgA and IgG antibodies against SARS-CoV-2 (SARS-CoV-2 ELISA, EUROIMMUN).

#### Brief case report 5: Male toddler with generalized febrile seizure (age group 1–3 years)

A male toddler was admitted late in March 2020 due to a generalized febrile seizure which spontaneously resolved after 4 min. The parents reported that he developed a low-grade fever up to 38.6°C several hours prior to the event. He also had moderate diarrhea with three to four pulpy bowel movements per day and vomited three times on the day of admission. The parents did not note any mucous or blood. On admission, clinical examination was unremarkable except for mild pharyngitis, intestinal hyperperistalsis, and mild dehydration. Differential blood count, liver enzymes, blood gasses, electrolytes, C-reactive protein, and urine analyses (except for ketone bodies) were all normal. Stool tests came back negative for *Rotavirus*, *Norovirus*, *Shigella*, *Campylobacter*, *Salmonella*, and *Yersinia*. The patient received intravenous fluids and was discharged 2 days later without fever in good clinical condition. At that time, the patient was diagnosed with mild gastroenteritis and secondary uncomplicated generalized febrile seizure. RT-qPCR and pyrosequencing (Figure S5) confirmed a SARS-CoV-2 infection.

#### Brief case report 6: Female toddler with multisystem inflammatory syndrome in children (MIS-C) (age group 1–3 years)

A female toddler was admitted to the hospital with altered general status and undulant fever. The initial physical examination revealed tonsillitis without any cardio-respiratory affections. Routine laboratory SARS-CoV-2 RT-qPCR tests were positive. Laboratory analyses revealed highly elevated C-reactive protein (24.0 mg/dL [normal <0.5]) with almost normal interleukin-6 levels at 55.2 pg/mL. Interestingly, no leukocytosis or lymphopenia were diagnosed. In the clinical course, symptoms reminiscent of Kawasaki-like disease included persistent fever, bilateral conjunctivitis, cheilitis, and a maculopapular exanthema. Furthermore, echocardiography exhibited enlargement of the left coronary artery and pericardial effusion (Figure S6). Cardiac-related blood parameters were within the normal ranges. Under the suspicion of multisystem inflammatory syndrome in children (MIS-C) associated with COVID-19 [14, 15], she was given intravenous gamma globulins (2 g/kg), prednisolone (2 mg/kg), and acetylsalicylic acid (50 mg/kg) at day 5 which resulted in rapid improvement of the girl’s general status. Apyrexia was achieved on day 7. On day 10 of hospitalization, SARS-CoV-2 RT-qPCR tests were negative. We did not perform pyrosequencing.

### Pre-selection of SARS-CoV-2 variant-of-concern candidates by pyrosequencing

Due to the incremental transmission of SARS-CoV-2 variants-of-concern, we note that the used strategy can uncover (Spike) P681H allowing the pre-selection of SARS-CoV-2 B.1.1.7 candidate specimens for deep sequencing. Whereas we did not see the P681H substitution in any of the analyzed pediatric specimens, we could detect it in 1 adult (Figure S5). To assess whether this specimen could be a carrier of the SARS-CoV-2 variant-of-concern B.1.1.7, we established a second pyrosequencing approach using primers amplifying a 513-bp amplicon that harbors the entire ACE2 receptor-binding domain (RBD) (Table S1). The amplicon amplifies faithfully under the same RT-qPCR conditions as described above (Figure S7). Using different sequencing primers (Table S1), we could not detect N501Y in the P681H sample, which is a key substitution for 3 variants-of-concern (i.e. B.1.1.7 [UK], B.1.3.51 [Africa], B.1.1.28p.1 [Brazil]). Also, not E484K could be detected. Remarkably, N501Y was seen in 2 other adult samples (Figure S7), but neither E484K nor P681H (data not shown).

## Discussion

### Numerous confounding factors influence the outcome of RT-qPCR in SARS-CoV-2 diagnostics

Whereas reverse transcription and subsequent quantitative PCR (RT-qPCR) are key methods for the detection of SARS-CoV-2 and local as well as global pandemic surveillance, it is well known that several confounding factors can lead to false-negative results. It seems clear that the time course of SARS-CoV-2 load in the days after infection massively influences the predictive value of the RT-qPCR tests. A recent study discovered a median false-negative rate as high as 38% (CI, 18 to 65%) even at the day of symptom onset, although concomitantly SARS-CoV-2 load seems to be close to its highest levels. Moreover, few days before and after symptom onset, the false-negative SARS-CoV-2 discovery rate by RT-qPCR seems to worsen dramatically [16]. Besides the time point of specimen sampling post-infection, heterogeneities in specimen sampling technique, transportation, storage conditions, nucleic acid purification, laboratory equipment, staff experience, and also RT-qPCR conditions might be among the most important factors influencing test qualities. Very early in 2020, the WHO started to disseminate the “Charité protocol” for the diagnostic detection of SARS-CoV-2 by RT-qPCR. Therein, a strategy for the combinatorial use of PCR primers (#name_F/#name_R) and TaqMan probes (#name_P) was described with amplicon targets in the SARS-CoV-2 RNA-dependent RNA polymerase (RdRP) gene as well as in the open reading frames (ORF) E and N (E_Sarbeco_F/_P1/_R; N_Sarbeco_F/_P/_R; RdRP_SARSr-F/_P2/_R) (Table S1) [13]. As of November 10, 2020, PubMed reported 976 citations for this article, suggesting the widespread use of the contained protocols for SARS-CoV-2 detection in association with the global attempt of pandemic surveillance and containment measures. However, several RT-qPCR assays are being used by clinical, research, and public health laboratories and some studies suggest that there could be relevant differences in the efficiencies, sensitivities, and specificities of the different amplicon targets under different laboratory conditions, in particular, for the RdRP_SARSr amplicon [17, 18]. Remarkably, RT-qPCR does not exclusively target genomic SARS-CoV-2 amplicons but (dependent on the selected primers) logically also can amplify the transcribed shorter subgenomic mRNAs (sgRNAs). At least two deep-sequencing studies on the transcriptomes of different coronaviruses (HCoV-229E and SARS-CoV-2) reported a dominant coverage of mapped reads towards the 3′-end of the SARS-CoV-2 genome, possibly due to an abundance of the sgRNAs [19, 20]. Notably, these sgRNAs encode conserved structural proteins with the Spike glycoprotein (S), the envelope protein (E), and the nucleocapsid protein (N) being among them. For the nonstructural RdRP, in contrast, the genomic (+)RNA itself becomes expressed by genome translation and ribosomal frameshifting [21]. The coverage of mapped reads was comparatively lower for the RdRP encoding ORF1b as well as the 5′-proximal ORF1a than for the sgRNA encoding 3′-end. However, it remains poorly understood so far how oscillations in the expression of SARS-CoV-2 sgRNAs might contribute to the observed variations in qPCR testing.

Therefore, we aimed to develop a confirmatory test for a stably expressed SARS-CoV-2 sgRNA amplicon target that can complement RT-qPCR strategies without disturbing established and automatable laboratory workflows. In particular, we intended to develop a pyrosequencing assay that would allow, subsequent to RT-qPCR, the categorical confirmation of SARS-CoV-2 infections in acute cases, where the clinical suspicion is high, but the SARS-CoV-2 infection cannot be ruled out by RT-qPCR alone. The proposed pyrosequencing approach does not negatively affect preceding RT-qPCR pipelines in SARS-CoV-2 diagnostics and can therefore add important value to RT-qPCR, where this method alone delivers conflicting results. Particularly, this can happen close to the detection limits of qPCR, practically C_T_ values ≫30. Frequently in laboratory practice, even negative samples exhibit curves crossing the threshold within a range between C_T_≈30 and C_T_≈35 complicating the reliable discrimination between PCR-negative and PCR false-negative specimens. However, with respect to all the above- and below-mentioned C_T_ values, we would like to point out that they can serve as viral load estimates only. Importantly, different PCR devices can report different C_T_ values for the same sample. For example, in some RT-qPCR assay manufacturers’ manuals, it is recommended that for the Corbett Rotor-Gene 6000 instrument (used in this study), C_T_ values ≥33 should be considered as negative calls, in contrast to C_T_ values ≥35 for other instruments (Qiagen RT^2^ Profiler Array System; https://www.qiagen.com/us/resources/resourcedetail?id=b3396407-ecb5-4656-ac5d-5ea7b83a397e&lang=en).

Theoretically, also PCR false-positives results could happen. This point is occasionally controversially discussed in society and by some scientists. But practically, we did not observe any case of false-positive diagnosis within the numerous confirmed SARS-CoV-2-positive specimens from clinical routine testing used during the entire phase of RT-qPCR/pyrosequencing development. Here, we have shown that pyrosequencing can be a powerful complementary method of specific and sensitive SARS-CoV-2 case confirmation, without affecting foregoing routine RT-qPCR. But even here, lower template concentrations led to the occasional occurrence of miscalled bases (missing bases: red triangle; excess bases: blue triangle) and gradual convergence of signal and noise peaks. Gradually, this led to increasingly misinterpreted peak heights particularly for nucleotide repeat motifs and lower complexity motifs. Whereas concomitantly, automated basecalling gradually failed to separate signal from noise, the SARS-CoV-2-specific sequence could be identified by manual inspection much longer. Manufacturer’s adaptations to the basecalling algorithm could lead to an improvement. Moreover, for each nucleotide position, we used an “open” pyrosequencing dispensation order (assuming N [i.e. C, G, A, or T] as possible nucleotides for each position) in order to detect variable positions. From the end users’ sides, a SARS-CoV-2-specific dispensation order could possibly lead to an improved automated basecalling sensitivity. However, being aware that any further sample treatment in laboratory routine would impair the necessary high-throughput testing, in particular, in the course of SARS-CoV-2 surveillance, we see the main application opportunities for combined RT-qPCR/pyrosequencing in research for retrospective studies, longitudinal studies of the course of COVID-19 cases, and studies on pathomechanisms, in particular, if they aim on systemic surveys of virus-host interaction.

### Several cases with unusual presentations of pediatric SARS-CoV-2 infections were uncovered

Exemplarily, after the implementation of the developed experimental pipeline, we conducted a field study in search of SARS-CoV-2-infected patients, who in spring and summer 2020 did not fulfill the contemporary indications for RT-qPCR testing. It appears that in the majority of cases, pediatric SARS-CoV-2 infections develop only very mild disease courses or have no symptoms at all [4]. In a very recent UK national cohort study on neonatal SARS-CoV-2 infection, 66 SARS-CoV-2-positive babies could be identified, who at the day of presentation exhibited hyperthermia, poor feeding, vomiting, coryza, other respiratory signs, and lethargy as the most common signs of infection or no signs of infection at all [3]. On the other hand, an unknown fraction of SARS-CoV-2 infections in childhood seem to fundamentally differ from adults and can be more heterogeneous in their presentation. The most striking example is multisystem inflammatory syndrome in children (MIS-C), a rare SARS-CoV-2-induced Kawasaki-like hyperinflammatory syndrome [14, 15]. Another recent study reports that children and adults can exhibit very different antibody responses upon SARS-CoV-2 infections across the clinical spectrum of associated diseases, which do not obligatorily match the adult COVID-19 spectrum [5]. Necessarily, the association of unusual symptoms with acute infections will contribute to our understanding about the heterogeneity of SARS-CoV-2-borne diseases in general and particularly in children. Using two large pediatric cohorts for a field study, we have successfully demonstrated in this study that combined RT-qPCR/pyrosequencing is a reliable tool that allows the faithful confirmation of mild or (almost) asymptomatic cases with SARS-CoV-2 infection close to the detection limits of RT-qPCR. Here, the determination of a SARS-CoV-2-specific sequence fragment can decisively help to improve reliability for cases where the discrimination between negative/false-negative RT-qPCR reports can be important. Whereas this might possibly be of subordinate importance for practical containment measures because low viral load could often be associated with low infectiousness, it can contribute to recognize the still probably underestimated extent of SARS-CoV-2 prevalence and associated mild or asymptomatic presentations. In particular, from a large cohort of children who did not fulfill the criteria for SARS-CoV-2 testing, we have retrospectively identified 5 cases of SARS-CoV-2-related symptoms. In opposite, only one single case of multisystem inflammatory syndrome in children (MIS-C) associated with SARS-CoV-2 was hospitalized in the course of the study. Carefully, with respect to the overall low number of SARS-CoV-2-positive cases in the local cohorts, we may nevertheless speculate that the numbers of unrecognized mild or symptomless pediatric cases might be relatively high. However, from our data, we feel incapable to estimate their contribution to SARS-CoV-2 transmission.

### Pre-selection of SARS-CoV-2 variant-of-concern candidates by pyrosequencing

Due to the incremental transmission of SARS-CoV-2 variants-of-concern, we note that the optional use of pyrosequencing can help to pre-select candidate specimens for deep sequencing approaches. Because there is currently an increasing demand for sequencing resources to monitor the transmission of SARS-CoV-2 variants-of-concern, this option can be a valuable tool in order to economize more laborious and expensive sequencing strategies. Therefore, it would be necessary to use selected biotinylated oligonucleotides in the preceeding RT-qPCR pipeline. In contrast, genotyping by pyrosequencing alone seems inprecise, since the solitary occurrence of N501Y or P681H is not sufficient for the detection of the currently emerging variants-of-concern.

## Conclusions

The proposed protocol might allow a specific and sensitive complementary confirmation of SARS-CoV-2 close to the detection limits of RT-qPCR. Combined RT-qPCR/pyrosequencing does not appear to negatively affect preceding RT-qPCR pipeline in SARS-CoV-2 diagnostics and could be optionally applied in routine to selectively inspect conflicting RT-qPCR results. Particularly in our study, the proposed workflow could be valuably exploited to identify and characterize several cases of previously unrecognized SARS-CoV-2-associated diseases in childhood, which can differ from adults and can be more heterogeneous in their presentation. Moreover, the RT-qPCR/pyrosequencing method could be used for the pre-selection of specimens for deep sequencing approaches in order to track the transmission of current variants-of-concern.

## Methods

### Sampling of pediatric and adult human specimens

Nasopharyngeal swabs or bronchoalveolar lavage specimens were collected at Helios University Hospital Wuppertal (North Rhine-Westphalia, West Germany) and Klinikum Kassel (Hessen, Central Germany) with approval of the Witten/Herdecke University ethics committee (covered by “CoronaKids” [No. 61/2020] and No. 160/2020 for the use of routinely sampled and confirmed specimens for RT-qPCR/pyrosequencing method establishment). Informed written consent was obtained from the legal guardians of the involved children. All work has been conducted according to the principles expressed in the Declaration of Helsinki.

### Storage and nucleic acids isolation

Specimens included nasopharyngeal swabs or bronchoalveolar lavage specimens, which underwent routine COVID-19 diagnostic testing. Pediatric cohort specimens were collected using brushes from the Gentra Puregene Buccal Cell Kit (100) (Qiagen, Cat. No. 158845) and then stabilized in 500 μL RNAlater™ (Thermo Fisher Scientific, Cat. No. AM7021). All specimens were stocked at −80°C. Total RNA was purified from 250 μL liquid specimen using 750 μL QIAzol lysis reagent (Qiagen, Cat. No. 158845) upon the manufacturer’s recommendations. RNA quality and quantity were assessed by microcapillary electrophoresis using the Small RNA kit (Agilent, Cat. No. 5067-1548) and the Agilent Bioanalyzer 2100 instrument.

Importantly, we determined that long-term storage (to date, up to 9 months) under these conditions allows unbiased RT-qPCR analyses. However, we observed that repeated freeze and thaw cycles of stored specimens as well as purified RNA affect sample quality and result in gradually increasing C_T_ values, similarly as reported by others [7].

### RT-qPCR

Quantitative analyses of SARS-CoV-2 (+)RNA from the human specimen was carried out combining reverse transcription and qPCR in a one-step protocol using Luna Universal Probe One-Step RT-qPCR Kit w/o ROX (New England Biolabs, Cat. No. E3007E) on a Corbett Rotor-Gene 6000 instrument. Primers and probes are described above and listed (Table S1). Per reaction, each primer and probes were used at 500 nM; 2 μL of purified template RNA was used for each single reaction volume of 20 μL. RT-qPCR conditions were as follows: Reverse transcription (RT) (60°C/30 min), initial denaturation and Hot Start *Taq* polymerase activation 95°C/2 min, cycling (36×[denaturation 95°C/15 s, extension 60°C/30 s]), and final extension 68°C/5 min. Thirty-six qPCR cycles were determined to largely avoid multiple unspecific byproducts and giving rise to sufficient amounts of biotinylated amplicon for subsequent pyrosequencing purposes. This amplicon could be used directly for pyrosequencing.

### Pyrosequencing

Pyrosequencing of the biotinylated single-stranded S-gene amplicon was performed on a PyroMark Q48 Autoprep device (Qiagen). The PyroMark Q48 Advanced CpG Reagents (4× 48) (Qiagen, Cat. No. 974002) including dNTPs, substrates, and enzymes are loaded to the assigned cartridges with volumes being adapted to the assay requirements. The designed approach theoretically allows to read a SARS-CoV-2 (+)RNA-specific sequence fragment of 55 nt. Further, the sequencing primer (S_pbc_-CoV-2-S) was added to the assigned cartridge in a predetermined volume; 10 μL of each RT-qPCR amplicon was loaded to each well of a PyroMark Q48 Disc, and then 3 μL PyroMark Q48 Magnetic Beads (300) (Qiagen, Cat. No. 974203) was added to each reaction. The sequencing reaction was initiated after denaturation and subsequent magnetic bead capture of the biotinylated single-stranded S-gene amplicon. The pyrosequencing software records the light signals corresponding to each well in the PyroMark Q48 Disc and saves the data graphically. Basecalled pyrograms were then manually inspected and analyzed for SARS-CoV-2 sequence verification.

## Supplementary Information

**Additional file 1: Table S1**. Oligonucleotides used for RT-qPCR and pyrosequencing. **Table S2**. Observed cases of pediatric SARS-CoV-2 infections (overview). **Figure S1**. Results of semi-quantitative PCR for the performance comparison using GITC-purified RNA or stabilized raw specimens as RT-qPCR substrates. Four confirmed SARS-CoV-2-positive specimens (p1 to p4) were used in combination with primer pairs targeting Orf E, RdRP, Orf N and S-gene amplicons in singleplex qPCR reactions. From GITC-purified templates, amplicons with the correct size could be amplified from all specimens. Notably, we observed lower molecular weight byproducts for Orf E PCR. From stabilized raw specimens, in contrast, the Orf E amplicon was not amplified from none of the four samples. With varying band intensity, amplicons with correct sizes were amplified from stabilized raw samples for all cases for RdRP and S-gene targets and in some cases for the Orf N target. However, for unknown reasons, band intensities appeared less stable when compared with purified RNA samples. Moreover, we observed lower molecular weight byproducts in some cases. **Figure S2**. Results of semi-quantitative PCR of the SARS-CoV-2 S-gene amplicon. **A.** After 36 PCR cycles and subsequent agarose gel electrophoresis the specific 162 bp amplicon corresponding to the SARS-CoV-2 protein S-gene was visible (red arrow). The gel was loaded with 8 RT-qPCR samples from confirmed SARS-CoV-2-positive cases (p1 to p8) and 1 negative control (n1). The occasional weak appearance of PCR byproducts (see p1) seemed to correlate with relatively low viral (+)RNA load in the specimen. **B.** An excess of PCR cycles (40x) leads to enrichment of unspecific lower and higher molecular weight byproducts in all samples from confirmed positive samples (p1 to p9) as well as negative controls (n1 to n3). These byproducts severely impaired the successive pyrosequencing leading to ambiguous results. **Figure S3**. Results of a triplex PCR approach for the simultaneous detection of amplicon targets for RdRP, Orf E and Orf N. For comparison, 14 SARS-CoV-2-negative samples (n1 to n14) and 11 confirmed SARS-CoV-2-positive specimens were analyzed by agarose gel electrophoresis. The simultaneous use of primers for RdRP, Orf E and Orf N amplicons leads to enrichment of unspecific lower and higher molecular weight byproducts in all samples, confirmed positives as well as negatives. Remarkably, in the fraction of negative samples bands of approx. 160 bp appear frequently. **Figure S4**. Example of overlapping RT-qPCR curves. Curves of positive controls are red, negative specimens are green, water control is purple. **Figure S5**. Pyrograms for 5 pediatric SARS-CoV-2-positive cases. Below the pediatric cases one adult example is shown, where the (Spike) P681H substitution was detected. **Figure S6**. Presentation of a female toddler (age group 1-3 years) with a Multisystem Inflammatory Syndrome in Children (MIS-C)/Kawasaki-like syndrome associated with SARS-CoV-2 infection. **A.** Echocardiography revealed an enlargement of the left coronary artery (LCA) with a pericardial effusion. **B.** Flow cytometric characterization of the peripheral mononuclear blood cells resulted in normal ranges of CD3+ T, CD4+ T helper, CD19+ B, and CD16+CD56+ natural killer cells. **Figure S7**. Amplicon size tests by microvolume electrophoresis using the Agilent Bioanalyzer and position of sequencing primers on the 513 bp fragment. **A.** L: DNA size ladder, lanes 1-2: 162 bp S-gene (PBC) amplicon, 3-4: 513 bp S-gene (RBD) amplicon. **B.** Position of a sequencing primer for analyses of residues 484, 486, 489, 493 and 494 (red shaded) as well as residue 501 (underlined). **C.** Top: pyrogram showing N501 in one specimen; bottom: pyrogram showing N501Y in one specimen

## Data Availability

Not applicable

## Abbreviations

(+)RNA: Sense polarity [single-stranded] ribonucleic acid
CI: Confidence interval
COVID-19: Coronavirus disease 2019
MERS: Middle East respiratory syndrome
C_T_: Cycle threshold
ORF: Open reading frame
RdRP: RNA-dependent RNA polymerase
RT-qPCR: Reverse transcription quantitative polymerase chain reaction,
SARS-CoV-1: Severe acute respiratory syndrome coronavirus 1
SARS-CoV-2: Severe acute respiratory syndrome coronavirus 2
sgRNA: Subgenomic RNA
*Taq*: *Thermus aquaticus*
