# Supplementary material for: Combined RT-qPCR and pyrosequencing of a Spike glycoprotein polybasic cleavage motif can uncover pediatric SARS-CoV-2 infections associated with heterogeneous presentation
Source: Mol Cell Pediatr. 2021 Apr 24;8:4. doi: 10.1186/s40348-021-00115-x (PMC8065314; doi:10.1186/s40348-021-00115-x)
Supplement: Supplementary file 1 — Additional file 1: Table S1. Oligonucleotides used for RT-qPCR and pyrosequencing. Table S2. Observed cases of pediatric SARS-CoV-2 infections (overview). Figure S1. Results of semi-quantitative PCR for the performance comparison using GITC-purified RNA or stabilized raw specimens as RT-qPCR substrates. Four confirmed SARS-CoV-2-positive specimens (p1 to p4) were used in combination with primer pairs targeting Orf E, RdRP, Orf N and S-gene amplicons in singleplex qPCR reactions. From GITC-purified templates, amplicons with the correct size could be amplified from all specimens. Notably, we observed lower molecular weight byproducts for Orf E PCR. From stabilized raw specimens, in contrast, the Orf E amplicon was not amplified from none of the four samples. With varying band intensity, amplicons with correct sizes were amplified from stabilized raw samples for all cases for RdRP and S-gene targets and in some cases for the Orf N target. However, for unknown reasons, band intensities appeared less stable when compared with purified RNA samples. Moreover, we observed lower molecular weight byproducts in some cases. Figure S2. Results of semi-quantitative PCR of the SARS-CoV-2 S-gene amplicon. A. After 36 PCR cycles and subsequent agarose gel electrophoresis the specific 162 bp amplicon corresponding to the SARS-CoV-2 protein S-gene was visible (red arrow). The gel was loaded with 8 RT-qPCR samples from confirmed SARS-CoV-2-positive cases (p1 to p8) and 1 negative control (n1). The occasional weak appearance of PCR byproducts (see p1) seemed to correlate with relatively low viral (+)RNA load in the specimen. B. An excess of PCR cycles (40x) leads to enrichment of unspecific lower and higher molecular weight byproducts in all samples from confirmed positive samples (p1 to p9) as well as negative controls (n1 to n3). These byproducts severely impaired the successive pyrosequencing leading to ambiguous results. Figure S3. Results of a triplex PCR approach for the simultane [file 40348_2021_115_MOESM1_ESM.docx]

**Table S1.** Oligonucleotides used for RT-qPCR and pyrosequencing.

| **Amplicon target** | **Oligo name** | **Sequence** | **Modification** | **Purpose** |
| --- | --- | --- | --- | --- |
| **S-gene** | S_pbc_-CoV-2-F | gcaggctgtttaataggggc | none | Forward primer |
|  | S_pbc_-CoV-2-R_BIO_ | accaagtgacatagtgtaggca | 5’-biotin-TEG | Reverse primer |
|  | S_pbc_-CoV-2-P | attggtgcaggtatatgcgctagttatc | 5’-HEX, 3’-BBQ-650 | Probe |
|  | S_pbc_-CoV-2-S | attggtgcaggtatatgcgctagttatc | none | Sequencing primer |
|  | S_RBD_-CoV-2-F | acaaatcgctccagggcaaa | none | Forward primer |
|  | S_RBD_-CoV-2-R_BIO_ | ggatcacggacagcatcagt | 5’-biotin-TEG | Reverse primer |
|  | S_RBD_-CoV-2-S(aa484) | cggtagcacaccttgtaatg | none | Sequencing primer |
|  | S_RBD_-CoV-2-S(aa501) | acaatcatatggtttccaaccc | none | Sequencing primer |
| **RdRP (Orf1b)** | RdRP_SARSr-F [12] | GTGARATGGTCATGTGTGGCGG | none | Forward primer |
|  | RdRP_SARSr-R [12] | CARATGTTAAASACACTATTAGCATA | none | Reverse primer |
|  | RdRP_SARSr-P2 [12] | CAGGTGGAACCTCATCAGGAGATGC | 5’-FAM, 3’-BBQ-650 | Probe |
| **Orf E** | E_Sarbeco_F [12] | ACAGGTACGTTAATAGTTAATAGCGT | none | Forward primer |
|  | E_Sarbeco_R [12] | ATATTGCAGCAGTACGCACACA | none | Reverse primer |
|  | E_Sarbeco_P1 [12] | ACACTAGCCATCCTTACTGCGCTTCG | 5’-FAM, 3’-BBQ-650 | Probe |
| **Orf N** | N_Sarbeco_F [12] | CACATTGGCACCCGCAATC | none | Forward primer |
|  | N_Sarbeco_R [12] | GAGGAACGAGAAGAGGCTTG | none | Reverse primer |
|  | N_Sarbeco_P [12] | ACTTCCTCAAGGAACAACATTGCCA | 5’-FAM, 3’-BBQ-650 | Probe |

**Table S2.** Observed cases of pediatric SARS-CoV-2 infections (overview).

| **Case** | **Age group** | **Description/diagnosis** | **Main symptoms** | **RT-qPCR/pyroseq** | **Follow-up** |
| --- | --- | --- | --- | --- | --- |
| Adolescent male | 13 - 17 years | inconspicuous course of SARS-CoV-2 infection | -slightly reduced general condition -sore throat -37.5°C body temperature | C_T_ (S gene): **25.47/25.33** C_T_ (ORF E): 23.78 C_T_ (ORF N): 27.47 C_T_ (RdRP): 31.23 pyroseq: confirmed SARS-CoV-2 | after 3 months: SARS-CoV-2 IgG |
| Pre-school boy | 4 - 6 years | short bowel disease history, suspected for sepsis | -high grade fever, no specific focus -elevated inflammatory markers improved after 1 day following intravenous antibiotics -blood culture positive for *Klebsiella pneumoniae* -at day 2 of inpatient care: facial, neck and upper limb oedema, vein distention in the upper chest and shortness of breath -CT-angiography of the chest confirmed superior vena cava (SVC) syndrome | C_T_ (S gene): **34.06** (repetitive mean) conspicuous curve shape pyroseq: confirmed SARS-CoV-2 | n.a. |
| Male 2ndary school child | 10 - 12 years | inconspicuous course of SARS-CoV-2 infection | -complaining about a ‘bump’ behind his left ear -non-tender swelling behind the left ear was interpreted as an enlarged lymph node | C_T_ (S gene): **35.56** (repetitive mean) conspicuous curve shape pyroseq: confirmed SARS-CoV-2 | n.a. |
| Female adolescent | 14 - 17 years | EBV-like disease temporally associated with SARS-CoV-2 infection | -1st hospitalization (late March 2020): high fever (40.2°C) -hepatomegaly and tonsillitis -confirmed acute Epstein-Barr-Virus infection -discharged: 3 days later without fever in good general condition -2nd hospitalization 9 days later: low temperature (38.4°C), general malaise, slightly increased liver enzymes -at that time symptoms suspected being EBV-associated -2 days later improved spontaneously, discharged home | C_T_ (S gene): **32.80** (repetitive mean) conspicuous curve shape pyroseq: confirmed SARS-CoV-2 | October 2020: SARS-CoV-2 IgG, IgA |
| Male toddler | 1 - 3 years | generalized febrile seizure diagnosed with mild gastro- enteritis and secondary uncomplicated generalized febrile seizure | -generalized febrile seizure, which resolved after 4 min -parents reported low grade fever (38.6°C) few hours prior event -moderate diarrhea, vomited 3x on day of admission, stool negative for Rotavirus, Norovirus, Shigella, Campylobacter, Salmonella and Yersinia -intravenous fluids, discharged 2 days later in good clinical condition | RT-qPCR positive pyroseq: confirmed SARS-CoV-2 | n.a. |
| Female toddler | 1 - 3 years | Multisystem Inflammatory Syndrome in Children (MIS-C) associated with COVID-19 | -altered general status and undulant fever -tonsillitis without any cardio-respiratory affections -C reactive protein (24.0 mg/dL [norm.<0.5])/almost normal interleukin-6 (55.2 pg/ml) -no leukocytosis or lymphopenia -persistent fever, bilateral conjunctivitis, cheilitis and a maculopapular exanthema -enlargement of the left coronary artery and pericardial effusion -intravenous gamma globulins (2g/kg), prednisolone (2mg/kg) and acetylsalicylic acid (50mg/kg) at day 5 resulted in rapid improvement of the general status | RT-qPCR positive, then (day 10 of hospitalization) RT-qPCR negative pyroseq: confirmed SARS-CoV-2 | n.a. |


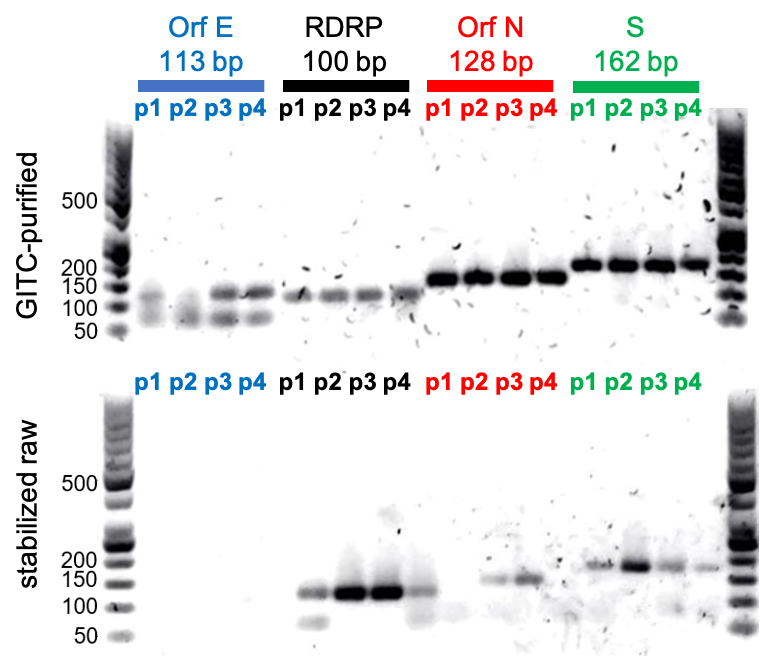


**Figure S1.** **Results of semi-quantitative PCR for the performance comparison using GITC-purified RNA or stabilized raw specimens as RT-qPCR substrates.** Four confirmed SARS-CoV-2-positive specimens (p1 to p4) were used in combination with primer pairs targeting Orf E, RdRP, Orf N and S-gene amplicons in singleplex qPCR reactions. From GITC-purified templates, amplicons with the correct size could be amplified from all specimens. Notably, we observed lower molecular weight byproducts for Orf E PCR. From stabilized raw specimens, in contrast, the Orf E amplicon was not amplified from none of the four samples. With varying band intensity, amplicons with correct sizes were amplified from stabilized raw samples for all cases for RdRP and S-gene targets and in some cases for the Orf N target. However, for unknown reasons, band intensities appeared less stable when compared with purified RNA samples. Moreover, we observed lower molecular weight byproducts in some cases.


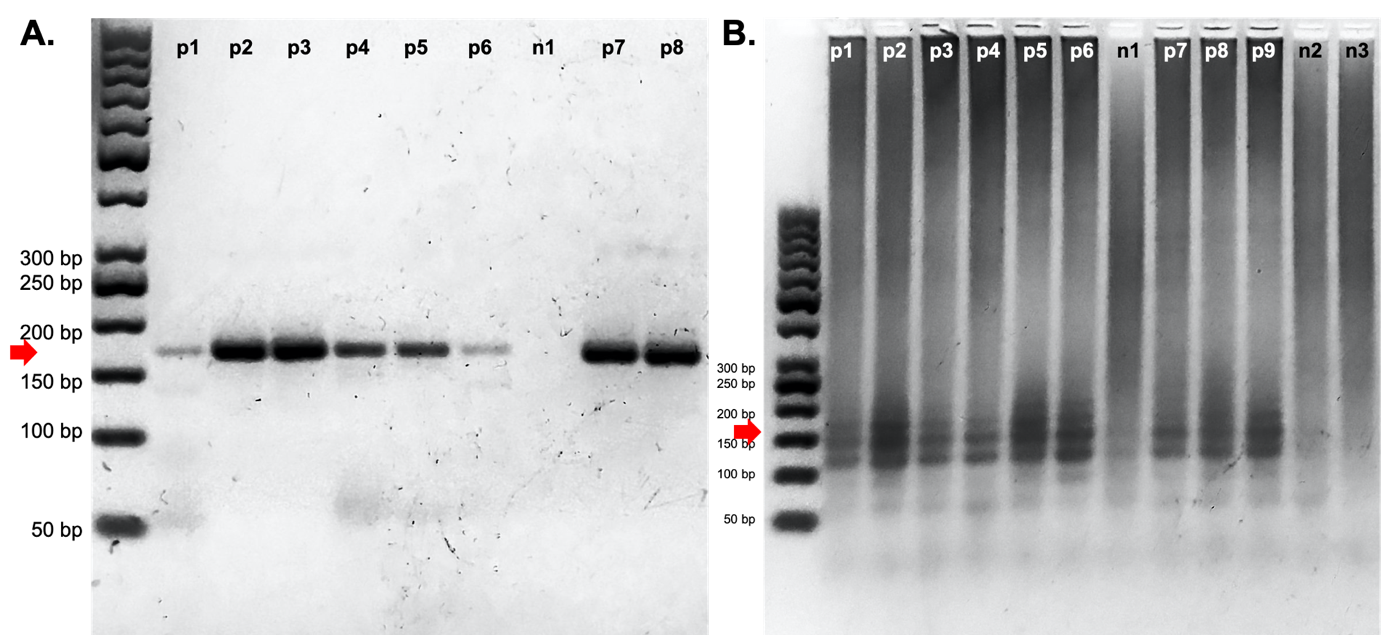


**Figure S2.** **Results of semi-quantitative PCR of the SARS-CoV-2 S-gene amplicon. A.** After 36 PCR cycles and subsequent agarose gel electrophoresis the specific 162 bp amplicon corresponding to the SARS-CoV-2 protein S-gene was visible (red arrow). The gel was loaded with 8 RT-qPCR samples from confirmed SARS-CoV-2-positive cases (p1 to p8) and 1 negative control (n1). The occasional weak appearance of PCR byproducts (see p1) seemed to correlate with relatively low viral (+)RNA load in the specimen. **B.** An excess of PCR cycles (40x) leads to enrichment of unspecific lower and higher molecular weight byproducts in all samples from confirmed positive samples (p1 to p9) as well as negative controls (n1 to n3). These byproducts severely impaired the successive pyrosequencing leading to ambiguous results.


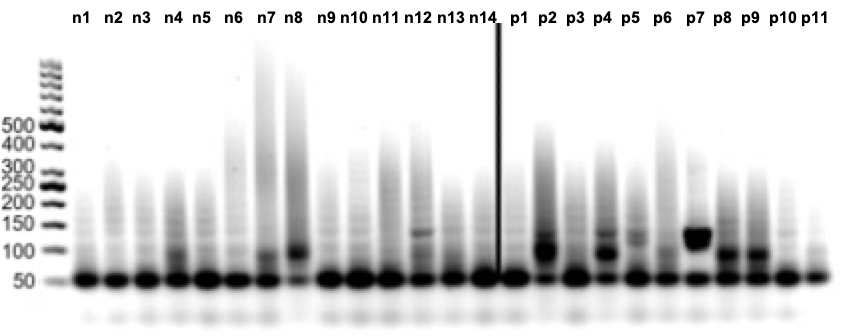


**Figure S3.** **Results of a triplex PCR approach for the simultaneous detection of amplicon targets for RdRP, Orf E and Orf N.** For comparison, 14 SARS-CoV-2-negative samples (n1 to n14) and 11 confirmed SARS-CoV-2-positive specimens were analysed by agarose gel electrophoresis. The simultaneous use of primers for RdRP, Orf E and Orf N amplicons leads to enrichment of unspecific lower and higher molecular weight byproducts in all samples, confirmed positives as well as negatives. Remarkably, in the fraction of negative samples bands of approx. 160 bp appear frequently.


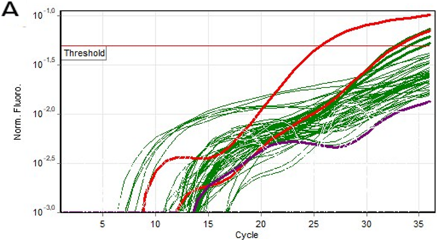


**Figure S4. Example of overlapping RT-qPCR curves.** Curves of positive controls are red, negative specimens are green, water control is purple.


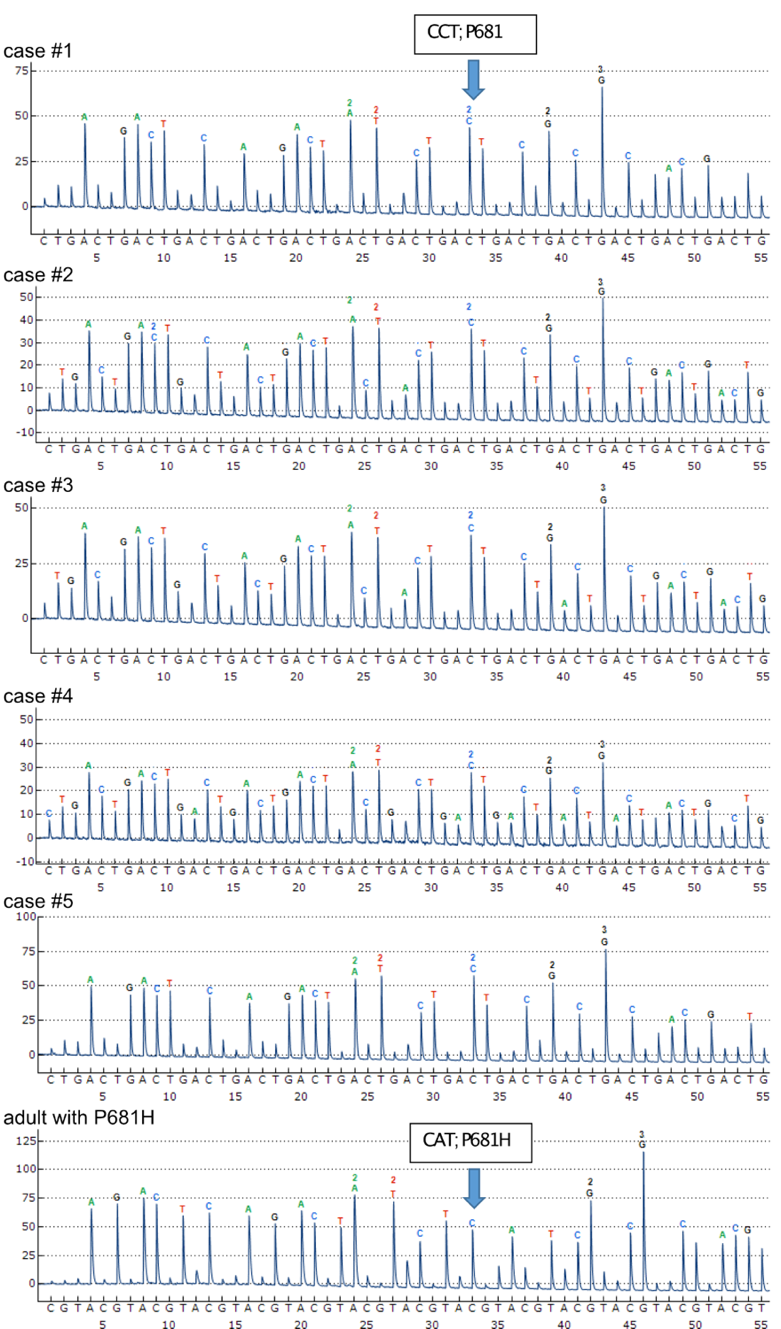


**Figure S5. Pyrograms for 5 pediatric SARS-CoV-2-positive cases.** Below the pediatric cases one adult example is shown, where the (Spike) P681H substitution was detected.


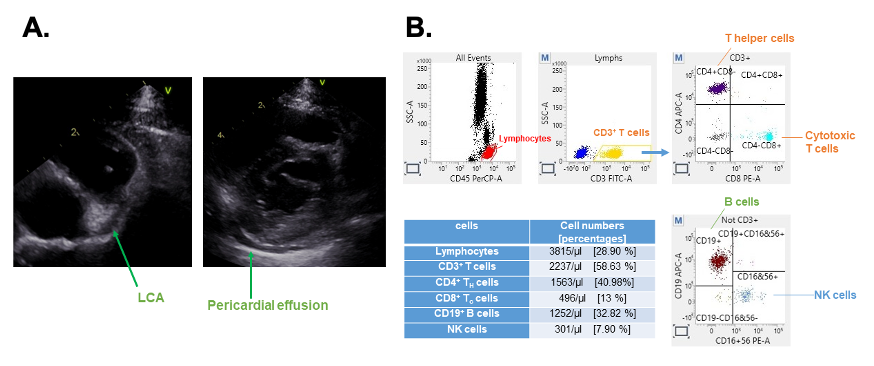


**Figure S6. Presentation of a female toddler (age group 1-3 years) with a Multisystem Inflammatory Syndrome in Children (MIS-C)/Kawasaki-like syndrome associated with SARS-CoV-2 infection. A.** Echocardiography revealed an enlargement of the left coronary artery (LCA) with a pericardial effusion. **B.** Flow cytometric characterization of the peripheral mononuclear blood cells resulted in normal ranges of CD3+ T, CD4+ T helper, CD19+ B, and CD16+CD56+ natural killer cells.


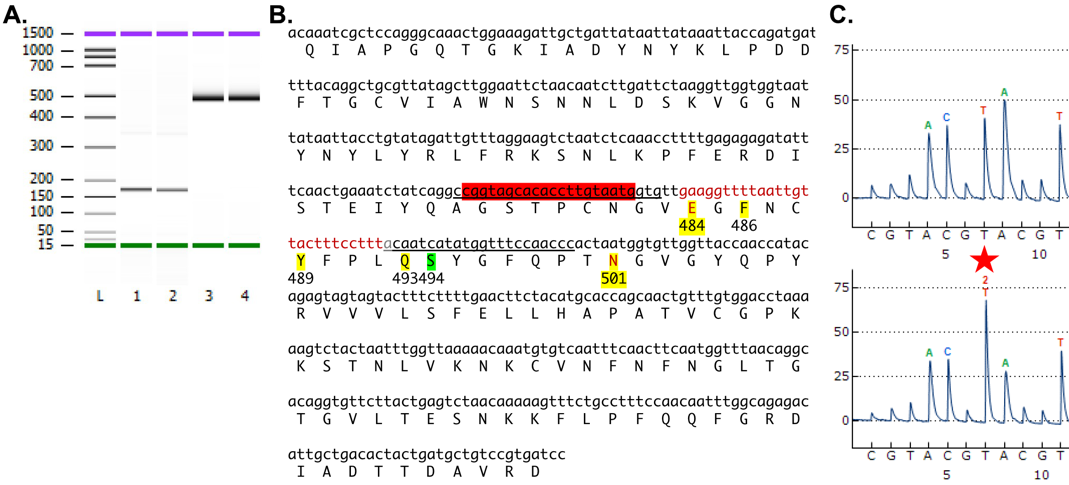


**Figure S7. Amplicon size tests by microvolume electrophoresis using the Agilent Bioanalyzer and position of sequencing primers on the 513 bp fragment. A.** L: DNA size ladder, lanes 1-2: 162 bp S-gene (PBC) amplicon, 3-4: 513 bp S-gene (RBD) amplicon. **B.** Position of a sequencing primer for analyses of residues 484, 486, 489, 493 and 494 (red shaded) as well as residue 501 (underlined). **C.** Top: pyrogram showing N501 in one specimen; bottom: pyrogram showing N501Y in one specimen.
